# Transcatheter Arterial Embolization for Atraumatic Splenic Rupture in TEE-negative Endocarditis: A Case Report

**DOI:** 10.5811/cpcem.1399

**Published:** 2024-02-13

**Authors:** Daniel P. High, Jose M. Acosta-Rullan, Danay Herrera, Mauricio Danckers, Daniel Heller, Daniel Zapata

**Affiliations:** * HCA Florida Aventura Hospital, Department of Pulmonary and Critical Care Medicine, Aventura, Florida; † HCA Florida Aventura Hospital, Department of Internal Medicine, Aventura, Florida; ‡ HCA Florida Kendall Hospital, Department of Pulmonary and Critical Care Medicine, Miami, Florida

**Keywords:** atraumatic splenic rupture, splenic artery embolization, endocarditis, case report

## Abstract

**Introduction:**

Spontaneous splenic rupture is an extremely rare complication of infective endocarditis.

**Case Report:**

We present a case of a 56-year-old immunocompetent female with porcine bioprosthetic mitral valve replacement, automated implanted cardioverter-defibrillator, and atrial fibrillation on apixaban who was found to have in-hospital atraumatic splenic rupture complicating infective endocarditis with *Haemophilus parainfluenza.* The rupture was treated successfully by endovascular embolization. Usual treatment with six weeks of antibiotics provided durable cure without further complication, and no surgical intervention was needed for either the valve or spleen.

**Conclusion:**

Transcatheter arterial embolization should be considered early in atraumatic splenic rupture. Relevant abdominal and cerebral imaging should be considered in all cases of suspected or confirmed infective endocarditis where unexplained symptoms are present.

Population Health Research Capsule What do we already know about this clinical entity?

*Atraumatic splenic rupture rarely complicates infection, malignancy, and inflammatory disease. Treatment follows pathways for more common traumatic splenic injuries.*

What makes this presentation of disease reportable?

*We describe definitive management of late, atraumatic splenic rupture complicating transesophageal echocardiogram-negative endocarditis using splenic embolization and antibiotics.*

What is the major learning point?

*Rare clinical entities can coexist and will not always be readily identifiable during an initial emergency department visit.*

How might this improve emergency medicine practice?

*Transcatheter splenic artery embolization should be considered early as a safe alternative to surgical management of atraumatic splenic rupture.*

## INTRODUCTION

The spleen, an encapsulated hematopoietic organ, is the most frequently injured visceral organ during abdominal blunt trauma.^1^ Patients who suffer from a splenic rupture often present with non-specific and subtle symptoms that may include sudden onset left upper quadrant abdominal pain (LUQ), abdominal distention, a rapid decrease in blood pressure and, if severe enough, alterations in mental status. While blunt trauma is the leading cause of splenic rupture, atraumatic splenic rupture (ASR) is a life-threatening condition that can occur spontaneously and in the absence of any apparent injury. Liu et al report ASR as a rare condition with an estimated incidence rate of 3.2% of all splenic ruptures, and Akoury et al points out that it is often misdiagnosed for other, more common causes of LUQ abdominal pain such as pancreatitis.^2^^,^^3^

Atraumatic splenic rupture carries a high mortality rate estimated at 12.2% because delays in diagnosis may lead to persistent internal bleeding, hemodynamic instability and, subsequently, death. Here we report our team’s emergent management of a rare case of *Haemophilus parainfluenzae* endocarditis-associated atraumatic splenic rupture. This subset of atraumatic splenic rupture is quite rare, and the use of endovascular rather than surgical treatment is also relatively novel. In a review of the literature we found no other similar cases treated this way.

## CASE REPORT

A 56-year-old immunocompetent woman with nonischemic cardiomyopathy with automated implanted cardioverter defibrillator (AICD), bioprosthetic mitral valve replacement secondary to rheumatic heart disease, paroxysmal atrial fibrillation on anticoagulation, and history of nephrolithiasis presented to the emergency department (ED) with LUQ pain. She denied any recent history of trauma. Laboratory data and computed tomography (CT) of the abdomen and pelvis with intravenous (IV) contrast were unremarkable. Secondary review of imaging done by an independent radiologist for publication confirmed a lack of splenic pathology. The patient was noted to have hematuria and discharged from the ED with suspicion for nephrolithiasis-associated renal colic from a stone that had already been passed out of the urethra. Five days later, she was asked to return to the hospital due to positive blood cultures growing *Haemophilus parainfluenzae*. On admission, she met modified Duke criteria for possible infective endocarditis (IE). She was started on IV ceftriaxone and continued on apixaban home regimen. A transesophageal echocardiogram (TEE) was performed without evidence of AICD lead or valvular vegetations, although mild mitral valve regurgitation was noted.

Persistent LUQ pain by hospital day three prompted a follow-up CT with oral and IV contrast of the abdomen and pelvis that revealed a large hemoperitoneum with a splenic hematoma measuring 15 × 9 × 15 centimeters. (Image) There was no definitive contrast blush, but active splenic hemorrhage was strongly favored by the reading radiologist. Her hemoglobin had dropped from 11.9 grams per deciliter (g/dL) to 7.1 g/dL (reference range: 11.2–15.7 g/dL). Emergent angiogram confirmed active extravasation at the inferior pole of the spleen during selective angiography of the splenic artery, and the splenic artery was embolized with seven coils and gel foam. The patient received three units of packed red blood cells. Apixaban had been held for more than 12 hours, and no reversal agent was indicated. Further intensive care unit and hospital course were uncomplicated, and the patient was discharged on day eight to complete a six-week course of IV ceftriaxone on home anticoagulation regimen.

**Image.**
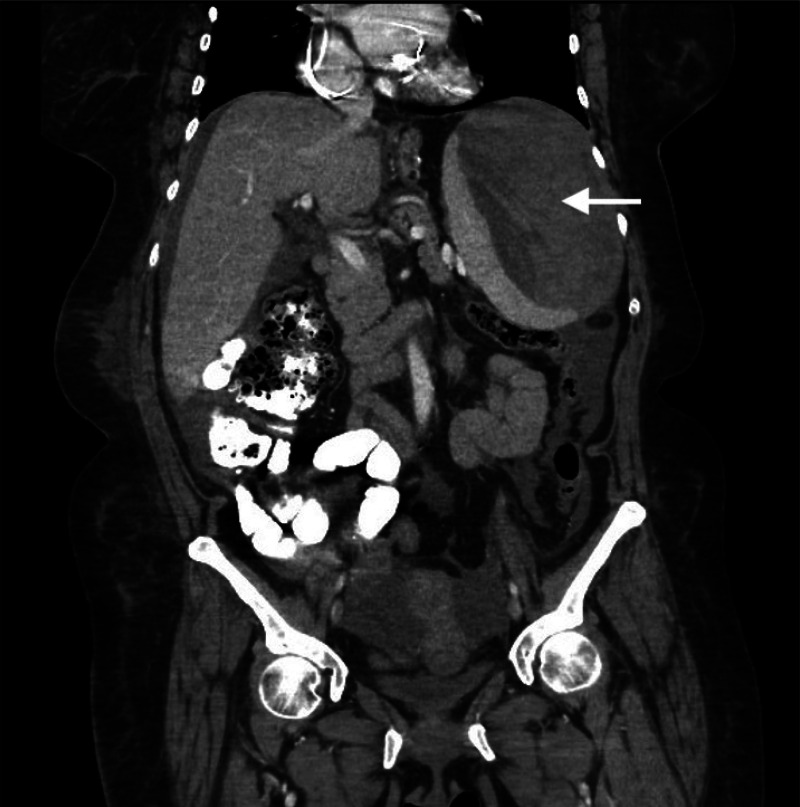
Contrast-enhanced coronal computed tomography of the abdomen demonstrating a large (15 × 9 × 15 centimeter) perisplenic hematoma (arrow) with concomitant hemoperitoneum due to atraumatic rupture.

She returned to a nearby hospital the next day for continued abdominal pain. No evidence of bleeding was seen at that time, but anticoagulation was held for a total of four months. No neurovascular complications were evidenced during this period nor recurrence of bleeding when anticoagulation was restarted. Surveillance cultures after antibiotic regimen completion were negative, and follow-up TEE did not show new findings.

## DISCUSSION

Atraumatic splenic rupture can be further divided into two categories: pathological (93%) and idiopathic (7%). The pathological etiologies include malignancies (30%), infectious (30%) inflammatory diseases (15%), medical treatments (10%), or mechanical causes (7%).^3^ Less commonly, ASR has also been reported as a fatal complication of IE (first reported case by Lake and Kevin in 1919).^4^ Here, we present a rare case of non-traumatic, spontaneous splenic rupture in the setting of *H parainfluenzae* endocarditis.

Spontaneous splenic rupture is an extremely rare complication of IE. While rare, missing the diagnosis may be catastrophic; therefore, a high index of suspicion is needed. In case series the condition carries a mortality rate of 15–58%.^6^ The mortality rate is variable depending on the etiology: neoplastic (21.4%), infectious (8.7%), inflammatory (9.5%), medical treatment (13%), mechanical (17%), and idiopathic (2%).^7^ Risk stratification and choice of intervention should be undertaken with these baseline mortality risks in mind.^7^

It is crucial to inquire regarding recent infections, surgical history, bleeding disorders, and use of anticoagulants, antiplatelets, or nonsteroidal anti-inflammatory drugs. There are three pathophysiological mechanisms of splenic rupture in endocarditis: 1) rupture of splenic abscess; 2) rupture of a mycotic aneurysm; and 3) rupture of a hematoma secondary to suppurating intrasplenic vessel, subcapsular dissection, and delayed capsular tear.^8^ Computed tomography is the current imaging modality of choice to diagnose splenic injury, but incorporating magnetic resonance imaging of the abdomen and brain may also be considered with concurrent IE as it has been shown to upgrade certainty of IE diagnosis or change treatment in 28% of IE cases.^10^

Surgical splenectomy is the most common intervention for ASR, although organ-preserving splenorrhaphy can also be done less commonly when appropriate; pediatric cases, for example, are more commonly managed conservatively or with organ-preserving treatment.^7^ Transcatheter arterial embolization (TAE) is becoming a more frequent treatment modality, particularly in cases involving anticoagulants, viral or protozoan etiology, or with active bleeding as this method may allow more rapid and safer hemostasis than surgery alone.^12^

The diagnosis of IE was made using five minor Duke endocarditis criteria for fever, mitral valve replacement, presumed splenic emboli, glomerulonephritis presumed based on hematuria without biopsy proof, and blood cultures not meeting major criteria. Circumstances precluded timely repeat blood cultures to obtain blood culture major criteria, and repeat cultures were taken after five days of antibiotics. There was unnecessary risk to the patient that precluded kidney biopsy to prove hematuria was related to glomerulonephritis given it would not have changed management. As splenectomy was not performed, no histopathology was performed on the spleen to prove embolism vs intrinsic splenic pathology. No repeat TEE was performed during the hospital course or that were available after discharge. In the setting of delayed splenic rupture, risk of ongoing infection was too high to justify a shorter antibiotic treatment course.

## CONCLUSION

When patients with possible or definitive IE have unexplained symptoms, there should be a low threshold of clinical suspicion for advanced imaging of the brain and abdomen to assess for embolic phenomena including splenic lesions. Such clinical information can prove crucial for identifying major complications in cases of TEE-negative endocarditis such as the one presented here. In addition, TAE can be a stabilizing and temporizing measure used in combination with critical care monitoring and intervention even if definitive surgical management may sometimes be needed later. Clinicians should consider TAE early in ASR and consider it a primary option during interdisciplinary decision-making to achieve favorable patient outcomes in this time-sensitive and high-risk clinical entity. We demonstrate here favorable and uncomplicated survival using TAE as definitive management in adjunct to usual antibiotic treatment of ASR complicating *H parainfluenzae* IE.
